# Baseline Diet Quality Is Related to Changes in the Body Composition and Inflammatory Markers: An Intervention Study Based on Resistance Training and Nutritional Advice

**DOI:** 10.1155/2021/6681823

**Published:** 2021-11-25

**Authors:** Daniel Barbosa Coelho, Lilian Maria Peixoto Lopes, Emerson Cruz de Oliveira, Lenice Kappes Becker, Guilherme de Paula Costa, Helen Hermana Miranda Hermsdorff, Fernanda Guimarães Drummond e Silva, Kelerson Mauro de Castro Pinto, André Talvani, Júlia Cristina Cardoso Carraro

**Affiliations:** ^1^Postgraduate Program in Health and Nutrition, Federal University of Ouro Preto, Ouro Preto, Minas Gerais, Brazil; ^2^Laboratory of Energy Metabolism and Body Composition, Department of Nutrition and Health, Universidade Federal de Viçosa (UFV), Viçosa, Minas Gerais, Brazil; ^3^Postgraduate Program in Health and Nutrition and School of Nutrition, Federal University of Ouro Preto, Ouro Preto, Minas Gerais, Brazil; ^4^Physical Education School and Inflammation Immunobiology Laboratory, Federal University of Ouro Preto, Ouro Preto, Minas Gerais, Brazil; ^5^Postgraduate Program in Health and Nutrition and Inflammation Immunobiology Laboratory, Federal University of Ouro Preto, Ouro Preto, Minas Gerais, Brazil

## Abstract

Habitual food intake and physical activity can affect chronic low-grade inflammation, which is common in the elderly, because of changes in the immune system and body composition. Thus, the present study proposes an evaluation of the influence of past eating habits on the effects of an intervention of resistance training plus dietary advice on the inflammatory profile of the elderly. We conducted an intervention study with 40 elderly people. The Revised Diet Quality Index (HEI-R) and the dietary total antioxidant capacity (dTAC) were calculated before the intervention based on a food frequency questionnaire validated to the elderly population. Participants were categorized according to the median of HEI-R and dTAC to assess the influence of the habitual diet quality on anthropometry and inflammatory markers (CRP, IL-8, CCL-2, and leptin) before and after the intervention. The 19-week intervention provided a long-term progressive resistance training associated with dietary advice focused on foods rich in compounds with anti-inflammatory and antioxidant properties. There was a greater reduction in weight, body mass index (BMI), and body fat (%) in the group with the lowest HEI-R and a greater reduction in the body fat (%) in the group with the lowest dTAC, indicating that the group that had a worse diet quality before the intervention responded better to it. The index HEI-R correlated negatively with Δweight and ΔBMI. dTAC correlated positively with Δmonocyte 1 chemotactic protein (CCL-2) and ΔC-reactive protein (CRP). In this scenario, elderly persons with bad habits can benefit from interventions to lifestyle change, while the better diet quality including dietary antioxidant sources can be useful to control weight and inflammatory biomarkers in this population.

## 1. Introduction

With aging, low-grade chronic inflammation is common once dysfunctions of the immune system cause an imbalance in the secretion of cytokines [1]. Furthermore, the increase in adiposity resulting from changes in body composition increases the secretion of proinflammatory cytokines by the adipose tissue [1–5].

Resistance exercises may modulate low-grade chronic inflammation, because this type of exercise reduces adiposity, increases the activity of antioxidant enzymes, and stimulates the secretion of anti-inflammatory cytokines [6–9].

However, the elderly's habitual food intake can also modulate low-grade chronic inflammation. The reduction in the consumption of saturated fats and simple sugars and the increase in the consumption of polyphenols, soluble fibers, and omega 3 fatty acids tend to reduce the plasma inflammatory markers [10–12].

Since these nutrients do not act in isolation, it is interesting to assess the quality as a whole in relation to inflammatory parameters. According to Previdelli et al. [13], the Revised Diet Quality Index (HEI-R) is an indicator of diet quality based on the energy density of daily portions from nine food groups, as proposed by the Food Guide for the Brazilian Population [14]. This index also includes the assessment of other nutrients that should be moderately consumed, such as sodium, saturated fats, and the AA fat component, referring to the energy resulting from solid fat, added sugar, and alcohol. Another method that makes available the evaluation of dietary quality is the dietary total antioxidant capacity (dTAC), which quantifies all antioxidants in the diet, such as polyphenols, carotenoids, ascorbic acid, tocopherols, and tocotrienols by means of the ferric reducing antioxidant power test (FRAP) [15, 16].

In this sense, the quality of the habitual diet could act synergistically with the resistance training and influence the inflammatory marker after the practice. The few existing studies on resistance training related to chronic inflammation in the elderly address physical exercise in isolation, without taking into account the possible influence of the quality of the diet [17–21], with positive results being found regarding inflammatory variables and anthropometric profile when the intervention with physical exercise was associated with dietary counseling in a study carried out by the present research group [22].

Following these investigations, the present study is aimed at assessing whether the quality of the basal diet using HEI-R and dTAC influences the effects of a resistance training intervention added to dietary counseling on anthropometry and profile inflammatory of the elderly.

## 2. Materials and Methods

### 2.1. Ethical Aspects

The present study was approved by the Research Ethics Committee on Humans of the Federal University of Ouro Preto (CAAE: 02761918.0.0000.5150), in compliance with the National Health Council's rules on research with human beings (Resolution 466/2012). The participants signed informed consent, which clearly described the freedom of participation, privacy, and access to information obtained during the research.

### 2.2. Study Design, Selection of Participants, and Eligibility Criteria

This is a prospective study that assessed data about anthropometry: weight, body mass index (BMI), waist circumference (WC), hip circumference (HC), waist-hip ratio (WHR), abdominal circumference (AC), arm circumference (ArmC), calf circumference (CC), and corrected arm muscle area (AMAc), and inflammatory profile: CRP, IL-8, CCL-2, and leptin before and after the intervention based on 19 weeks of resistance training associated with dietary advice focused on foods rich in compounds with anti-inflammatory and antioxidant properties. Subjects were categorized according to HEI-R and dTAC median before the intervention to assess the influence of the previous habitual diet on the results of the intervention.

The sample size (*n* = 43) was calculated by comparing paired groups [23] considering a 95% confidence level and an 80% power.

The inclusion criteria to be 60 years old or older and to perform medical and physical evaluation to certify that they have health conditions that allow them to practice physical activity (resistance training). The exclusion criteria were to have diseases such as uncontrolled diabetes, uncontrolled arterial hypertension, uncontrolled dyslipidemia, heart disease, osteoporosis, lung disease, or any other that would prevent them from performing resistance training and to obtain a percentage of attendance in the training program below 70%, nonadherence to dietary guidelines, being a smoker, or on a diet to lose weight in the last 3 months.

This study is a continuation of the investigation of this research group and presents different variables and new interpretations of the data generated by this intervention with this group of volunteers.

### 2.3. Dietary Analysis

To determine diet quality (HEI-R and dTAC), a semiquantitative food frequency questionnaire validated for the elderly Brazilian population by Henn [24] was applied before the intervention.

Subsequently, we obtained the nutritional analysis of each participant using Microsoft® Excel and the Table for Assessment of Food Consumption in Home Measures [25], Table of Food Composition [26], and Table of the United States Department of Agriculture [27], in this order of priority.

The HEI-R score was obtained by summing the scores of adequacy components (total fruits, whole fruits, total vegetables, dark green and orange vegetables, total cereals, whole grains, milk and dairy products, meat/eggs/legumes, and oils) and moderation components (sodium, saturated fat, solid fat, alcohol, and added sugar), as proposed by Previdelli et al. [13].

The dTAC was obtained by multiplying the amount of food or drink by the corresponding FRAP value and summing all food sources present in the diet [15]. The dTAC values were expressed in mmol per 100 grams of food (mmol/100 g) and adjusted by energy by the residual method [28], as well as the other nutrients.

Participants were categorized according to the median HEI-R and dTAC to assess the influence of the habitual quality diet on the results of the intervention (CRP, IL-8, CCL-2, and leptin).

### 2.4. Intervention

The intervention consisted of a long-term progressive resistance training associated with nutritional advice for 19 weeks. The long-term progressive resistance training happened twice a week on nonconsecutive days and lasted one hour. During two weeks, there was familiarization with the exercises and equipment using a minimum load. Then, the 1-MR prediction test was applied. In the 1^st^ and 2^nd^ weeks, the participants trained with 60% of the 1-MR load; in the 3^rd^ and 4^th^ weeks, with 70%; and in the 5^th^ and 6^th^ weeks, with 80% of the 1-MR load, and with 85% of the 1-MR load, they trained with 80% load from the 7^th^ week onwards. The number of repetitions proposed was 12 to 15 repetitions in the percentage of 60% of 1-MR, 10 to 12 repetitions for 70%, and six to eight repetitions for 80 and 85% of the 1-MR load [29, 30].

The nutritional advice was based on a list of foods rich in compounds with anti-inflammatory and antioxidant properties according to scientific studies [31–36]. In nutritional counseling, we advised participants to increase the consumption of prebiotic foods (soluble fibers); antioxidants, such as polyphenols; and foods rich in omega 3 fatty acids, as well as to reduce the consumption of saturated fatty acids and simple sugars. A nutritionist gave the elderly the advice at the beginning of the training program in printed and verbal form and repeated it verbally once a week. Adherence to nutritional guidelines was assessed using 24-hour food records (before and after the intervention).

### 2.5. Anthropometry

Weight was measured using a portable Tanita® scale, with capacity of 150 kg, where the participant was weighed barefoot and wearing light clothing. Height was measured using the Sanny® portable anthropometer, spanning 115 to 210 cm [37]. The BMI was calculated as weight/height^2^ to classify the nutritional status of the elderly, according to Lipschitz [38].

To measure the circumferences, a flexible and inelastic measuring tape was used (precision of 1 mm), and the circumferences were measured as described by Lohman et al. [37]. The AMAc was calculated using the equation by Heymsfield et al. [39], and the waist-hip ratio (WHR) was obtained by dividing WC by HC [40]. Skinfolds were measured using a Cescorf® adipometer with a sensitivity of 0.1 mm, reading range of 85 mm, and pressure of 10 g/mm^2^ [37]. The percentage of body fat (BF) was estimated by the sum of four skinfolds, according to the equation of Durnin and Womersley [41].

### 2.6. Blood Collection and Inflammatory Profile Analysis

A trained professional collected the blood using intravenous puncture and vacuum system in medial or cephalic veins in the anterior area of the arm. Then, the blood samples were centrifuged at 3,000 rpm for ten minutes where the blood components were separated. The aliquots were pipetted and stored.

The serum CRP assessment was performed using the turbidimetric inhibition immunoassay with a specific kit for the Cobas Integra 400 Plus equipment (Roche®). The biomarkers IL-8, CCL-2 (sensitivity from 8 to 1,000 pg/mL), and leptin (sensitivity from 63 to 4,000 pg/mL) were performed using the ELISA method with specific kits from PeproTech® and following the manufacturer's protocol.

### 2.7. Statistical Analyses

Data were tabulated, and normality was assessed using the Shapiro-Wilk test. The results are expressed as mean and standard deviation in the case of normal data, or median (minimum and maximum) if not parametric.

Participants were categorized according to the median of HEI-R and dTAC (total *n* = 40, lowest HEI‐R = 20, highest HEI‐R = 20 and lowest dTAC = 20, highest dTAC = 20). The differences between the unpaired groups (lower and upper HEI-R and dTAC) were assessed by Student's *t*-test or Mann–Whitney test. The differences between the paired groups before and after the intervention were assessed using the paired *t*-test or the Wilcoxon test according to the data distribution (*n* = 40). The correlations between continuous variables were determined by the Spearman correlation (*n* = 40).

The analyses related to the adjustment of nutrients in the usual food intake were performed using the STATA® software, version 13.0 (*n* = 40). Tests of normality and differences between groups were performed using the GraphPad Prism® software, version 6.0. For all analyses, a significance level of 5% was adopted.

## 3. Results

The present study had the participation of 40 elderly people, with an average age of 63.9 ± 3.8 years, and among them, 26 (65%) were women. Among the elderly participants in the present study, 8 (20%) had type 2 diabetes, 21 (53%) had arterial hypertension, 8 (20%) had dyslipidemia, and 3 (7%) had type 2 diabetes, arterial hypertension, and dyslipidemia.

**Figure 1:**
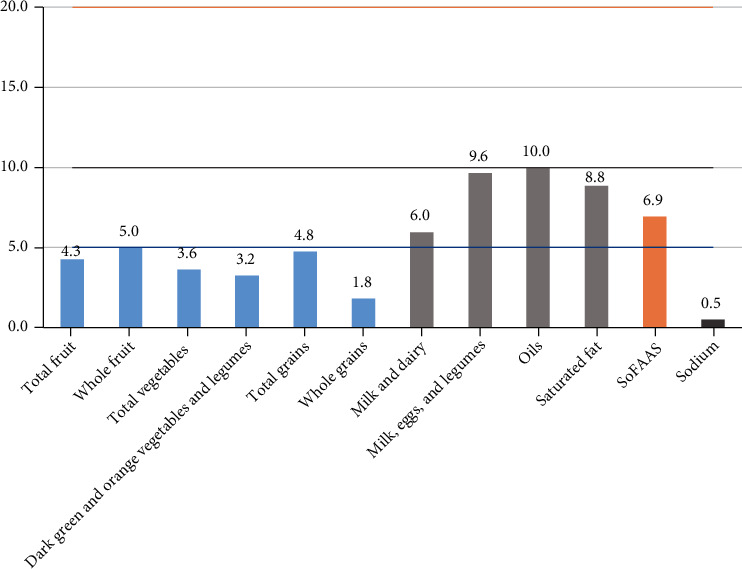
The components of the Revised Health Eating Index (HEI-R), where the higher the score means the better the quality of the diet. Maximum score for the component's total fruits, whole fruits, total vegetables, dark green and orange vegetables, total cereals, and whole grains represented by the blue line (5 points). Maximum score for the components milk and dairy products, meat, eggs and legumes, oils, saturated fat, and sodium represented in the gray line (10 points). Maximum score for the component of calories from solid fat, alcohol, and added sugar represented by the orange line (20 points). For the moderation components (sodium, saturated fat, and calories from solid fat, alcohol, and added sugar), higher scores correspond to lower consumption. For the other components classified as adequacy components, the higher the consumption, the higher the score. SoFAAS: calories from solid fats, alcoholic beverages, and added sugars.

The average HEI-R score was 64.6 ± 6.3, the average dTAC value was 14.6 ± 5.4 mmol/day, and the average energy was 1,878.7 ± 369.5 kcal/day.  Figure 1 presents the score of the components of the HEI-R.

By categorizing participants according to the median HEI-R and dTAC, in the group with the lowest HEI-R, there was a reduction in weight, BMI, CP, BF percentage, and leptin and a significant increase in AMAc. On the other hand, in the higher HEI-R group, there was a significant reduction in CP, BF percentage, CCL-2, and leptin and an increase in ArmC, AMAc, and CC. By comparing the differences between the smaller and larger HEI-R groups, there is a difference only in weight, BMI, and BF percentage between groups, indicating a more marked improvement in the smaller HEI-R group (Table 1).

When categorized according to dTAC in the usual diet, in the smaller dTAC group, there was a reduction in WC and BF percentage and an increase in AMAc. In contrast, in the larger dTAC group, there was a reduction in WC, WHR, BF percentage, CCL-2, and leptin and an increase in AMAc. By comparing the differences between the smaller and larger dTAC groups, there is a difference only in BF percentage, whose reduction was greater than that in the group with the lowest dTAC (Table 2).

**Figure 2:**
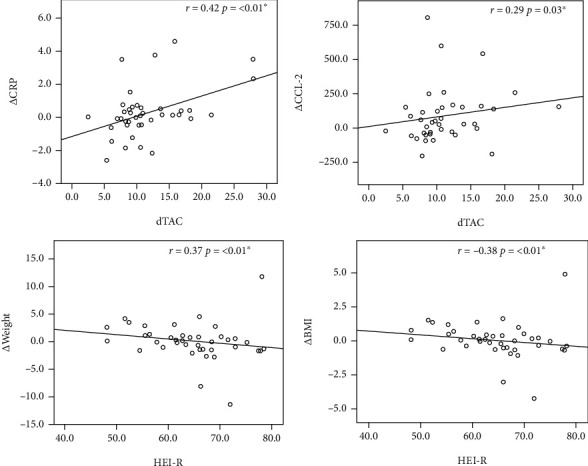
Correlations between quality and antioxidant capacity of the diet and Δ of anthropometric and inflammatory variables. Δ: delta (initial-final). HEI-R: Revised Health Eating Index; dTAC: dietary total antioxidant capacity; BMI: body mass index; CRP: C-reactive protein; CCL-2: monocyte chemotactic protein 1.

By assessing the usual intake in relation to Δ of the studied variables, there was a negative correlation between HEI-R and Δweight and ΔBMI, indicating that the higher the HEI-R, the smaller the difference in weight after the intervention. Whereas for dTAC, there was a positive correlation between dTAC and ΔCRP, and ΔCCL‐2, evidencing that the higher the dTAC, the greater the reduction in these inflammatory biomarkers (Figure 2).

## 4. Discussion

Resistance training can help reduce chronic inflammation in the elderly, especially when combined with dietary advice [22]. However, the available studies have not addressed a possible concomitant influence of habitual diet quality and other lifestyle factors on inflammatory profile [17–21]. Given that the elderly's eating habits are directly related to low-grade chronic inflammation [10–12], the present study evaluated the influence of past diet quality on the effects of resistance training associated with dietary advice on chronic low-grade inflammation for the elderly. It is important to highlight that other factors can affect the inflammatory profile, such as smoking and alcohol consumption; however, the habit of smoking was considered an exclusion factor in this study, and alcohol consumption was evaluated as one of the components of HEI-R.

Although there is not a cutoff, elderlies have shown a moderated score for HEI-R. The components of the HEI-R that have the lowest scores were whole grains (high in fiber), sodium and calories from saturated and trans fats, alcohol, and added sugar. Assumpção et al. [42] assessed the diet quality by the HEI-R of 1,509 elderly people in the city of Campinas, SP, Brazil, and reported a close value (62.40) to that found in the present study (64.68). This methodology has been recommended by different studies. Previdelli et al. [13] reported the applicability of HEI-R to estimate the habitual food intake at different stages of life, including the elderly. Andrade et al. [43] also showed the validity and reliability of the index to assess the diet quality in Brazil.

Although HEI-R is based on the recommendations of the previous Food Guide for the Brazilian Population [14], this index is still valid for the present research as it is a reliable way of measuring quantitatively the food consumption through recommendations of daily portions of food groups that are not present in the current guide (Food Guide for the Brazilian Population [14]). In addition, the absence of a diet quality index based on the current food guide justifies the use of HEI-R in this study. In general, the score obtained before the intervention revealed that elderly participants should increase the consumption of dietary fiber and reduce the consumption of saturated fats and sodium to further improve the diet and the inflammatory profile.

Studies have shown that short-chain fatty acids produced by fermentation of dietary fibers have an anti-inflammatory action, once they inhibit the nuclear factor kappa B (NF-*κ*B), blocking proinflammatory cytokines (IL-6, IL-8, IL-12, and TNF) and releasing and increasing the expression of anti-inflammatory cytokines, such as IL-10 [44–46]. Wannamethee et al. [47] reported that fiber consumption was inversely proportional to levels of CRP and IL-6 in the elderly, indicating that the lower the dietary fiber intake, the higher the concentrations of these inflammatory biomarkers. Similarly, Butcher and Beckstrand [48] pointed out in their review a negative correlation between fiber consumption and the inflammatory biomarker, CRP. The findings of the present study are corroborated by the aforementioned studies, since there was a positive correlation between dTAC and CRP (Figure 2).

As addressed in this study, the Brazilian Society of Cardiology [49] recommends moderating the intake of foods of animal origin (fatty meats, milk, and dairy products), as well as coconut and palm oil, in order to reduce the consumption of saturated fatty acids. According to Gaesser et al. [50], the high consumption of saturated fats (myristic, palmitic, and stearic acids) is associated with high concentrations of low-density lipoprotein (LDL-c) and a consequent increased risk of developing cardiovascular diseases. LDL-c is directly involved in the atherosclerotic process due to its oxidation and attraction of monocytes and neutrophils responsible for releasing inflammatory cytokines, such as CCL-2 and IL-8. Another issue is that excess of saturated fatty acids increases oxidative stress in the endoplasmic reticulum and generates reactive oxygen species that stimulate the NF-*κ*B pathway, leading to the production of inflammatory cytokines by adipocytes [51, 52].

Although the sodium consumption of participants was close to 2 g/day, as recommended by the Brazilian Society of Cardiology [53], it is emphasized that the usual intake was above the daily needs for the age group 51-70 years (1.3 g/day), as recommended by the Institute of Medicine [54]. This deserves attention, since studies have shown that high sodium concentrations can increase oxidative stress and activate NF-*κ*B, accentuating inflammatory processes associated with the progression of cardiovascular diseases and diabetes [55]. These statements corroborate the findings of the present study in which the groups with the best dTAC and the highest HEI-R showed a decrease in CCL-2 in pre- and postsituations. Still in this scenario, the Brazilian Society of Cardiology [53] shows that a daily sodium intake above 2 g/day is related to an increase in the prevalence of arterial hypertension.

Although the literature shows a link between some components of the HEI-R and inflammation, in the present study, there was no correlation between the HEI-R score and Δ of the studied inflammatory biomarkers (CRP, IL-8, CCL-2, and leptin). This suggests that the diet quality assessed by the HEI-R before the intervention did not influence changes in inflammatory markers after the intervention.

However, the HEI-R had a positive influence on anthropometric parameters given that, when categorizing the participants into lower and higher score groups, there was a significant reduction in weight in the lower HEI-R group, which was reflected in the BMI. Corroborating this result, there was a negative correlation between HEI-R and the Δweight, indicating that the difference in weight was greater in the lower HEI-R group. In addition, the reduction in the percentage of BF was more marked in the lower HEI-R group and in the lower dTAC. The methods used to assess body composition in the present study are widely used and validated. Although other methods may be more accurate in assessing regional fat distribution and body composition, they are of little use in clinical practice, given their drawbacks such as high cost and complexity and, in the case of some, exposure to radiation [40, 41]. These results suggest that the group that had a worse diet quality before the intervention responded better to the dietary advice and resistance training, given that, once this group initially presented a poorer diet quality, greater changes in eating habits may have occurred, resulting in a more negative energy balance.

In the present study, the average dTAC value was 14.6 mmol/day. Similar to this finding, Kobayashi et al. [56] investigated the diet of Japanese elderly women and reported a value of 13.6 mmol/day. However, Mekary et al. [57] showed a lower value in elderly Americans (10.8 mmol/day), which is justified by the dietary pattern of that country. Studies on elderly Brazilians that address the determination of dTAC are scarce. Souza [58] found a value of 11.9 mmol/day, being coffee and tea the foods with the greatest contribution to dTAC, followed by vegetables and fruits, in elderly people in the city of Viçosa-MG/Brazil.

dTAC assesses all antioxidants in the diet, such as polyphenols, carotenoids, ascorbic acid, tocopherols, and tocotrienols. The literature is controversial about the use of dTAC, since the bioavailability of antioxidants in the diet is not yet known [15]. However, recent studies suggest that there is a synergistic effect between the food components released by the digestion process that enhances antioxidant compound bioaccessibility and absorption [59–62]. These components may act as carriers of antioxidant compounds, through the gastrointestinal tract, protecting them from oxidative reactions and maintaining their stability [59, 61]. Also, some authors have shown a positive correlation between dTAC and total antioxidant capacity in the plasma [63, 64]. In addition, studies in the elderly population have also shown that the higher the dTAC, the lower the risk of developing depression, frailty, heart failure, and ischemic stroke [56, 65–67].

During lifespan, oxidative stress increases due to the accumulation of harmful effects caused by reactive oxygen species and the reduction of antioxidant defenses. In this context, the diet should contain antioxidants that protect the body from increases in production of free radicals at an old age [68], as inferred in the present study. These statements agreed with the findings of a positive correlation of the dTAC of the diet and the variation of the inflammatory markers of the present study.

In this sense, the consumption of a diet with a high antioxidant capacity tends to improve the inflammatory profile since the antioxidant compounds activate the Nrf2 pathway, which increases the activity of antioxidant enzymes. As a result, there is a reduction in reactive oxygen species and a consequent inhibition of NF-*κ*B, responsible for the expression of proinflammatory cytokines [69]. In the present study, there was a positive correlation between dTAC and the Δ of inflammatory biomarkers CRP and CCL-2, evidencing that the higher the dTAC, the greater the reduction of these parameters after the intervention. In this sense, Valtuena et al. [70] and Brighenti et al. [68] also showed that the higher the dTAC, the lower the plasma concentrations of CRP.

It has already been shown that a resistance exercise program of long duration and progressive intensity associated with dietary guidance is able to reduce chronic inflammation in the elderly [22]. However, this current study clarifies that groups with poor dietary quality benefit most from this combination of exercise and nutritional counseling. This clarification can be interpreted as a motivational factor for the population who have bad eating habits and who have difficulty in behavioral change. In addition to these points, attention is drawn to the need to work together with different health professionals for a multidisciplinary approach to elderly people.

It has been identified that the inflammatory condition is greater with advancing age, and this occurs mainly in women [71, 72]. The exacerbation of the inflammatory condition in women is also related to the genetic profile [73] and to the body composition [74]. This difference between the inflammatory profile of men and women is well founded in the literature, but when several lifestyle parameters were considered to be covariates, there was no difference between genders [75].

It is observed that adipose tissue contributes to the production of inflammatory cytokines and is present in conditions of diseases with metabolic impairment such as dyslipidemia and type 2 diabetes [74]. So, it should be noted that body composition differs between men and women. Men have more visceral adipose tissue and greater inter- and intramuscular adipose tissue and pericardial adipose tissue, which is associated with greater cardiometabolic risk. Women, on the other hand, have more subcutaneous adipose tissue and potentially more brown adipose tissue. This female pattern of fat distribution is associated with improved cardiometabolic risk with similar BMI [74].

It should be noted that even with the differences in inflammation parameters related to age and body composition between men and women previously reported, the present study did not compare these variables between these groups. Although the comparison between the variables was made mainly between the pre- and postintervention situations and the number of volunteers in each group should be reduced with this comparison, the failure to perform this treatment should be considered a limitation of the study.

Another limitation of this study is the lack of a control group to assess the separate effects of nutritional counseling and the resistance training program, but the main objective of the present study was to identify the synergistic effect between exercise and diet. In addition, the study design may have been influenced by family history, educational level, and economic status. However, the difficulties and applicability of the evaluation in humans with normal routines are highlighted, which characterizes the high external validity of the study. Another limitation is the evaluation only of elderly people with this intervention, but in addition to this being the public to be studied as previously mentioned, there was a restriction of financial resources and a greater number of researchers in the field.

## 5. Conclusion

The present study shows a positive influence of diet quality (HEI-R and dTAC) on the effects of the intervention on anthropometric parameters weight, BMI, and BF percentage. dTAC correlated positively with the reduction in CCL-2 and CRP. Therefore, simple interventions in the planning of assistance programs for the elderly should include, in addition to resistance exercises, dietary advice since the quality and antioxidant capacity of the habitual diet can collaborate (in different ways) with healthy aging.

## Figures and Tables

**Table 1:** Anthropometric and inflammatory variables, according to the usual HEI-R and 19-week intervention.

≤HEI-R (*n* = 20)					>HEI-R (*n* = 20)				*p* values ª
Variable	Baseline	Final	*p* values	Δ	Baseline	Final	*p* values	Δ	
Weight (kg)	75.2 ± 11.9	74.4 ± 11.4	0.03^∗^	0.86	68.8 ± 14.9	69.5 ± 14.7	0.55	-0.61	0.04^a^
BMI (kg/m^2^)	29.0 ± 4.9	28.7 ± 4.7	0.03^∗^	0.33	26.2 ± 3.6	26.5 ± 3.3	0.58	-0.22	0.03^a^
WC (cm)	92.1 ± 7.7	89.2 ± 8.9	0.01^∗^	2.94	86.6 ± 11.4	83.8 ± 10.5	<0.01^∗^	2.85	0.60
HC (cm)	101.3 ± 10.7	102 ± 8.8	0.70	-0.70	98.4 ± 8.7	98.7 ± 8.4	0.57	-0.35	0.99
WHR	0.91 ± 0.08	0.87 ± 0.08	0.07	0.04	0.88 ± 0.08	0.84 ± 0.06	0.05	0.04	0.79
AC (cm)	96.4 ± 12.4	96.4 ± 11.2	0.92	0.09	90.1 ± 12.0	89.2 ± 13.2	0.61	0.86	0.64
AP (cm)	30.1 ± 3.6	31.1 ± 3.3	0.07	-1	30.2 ± 4.4	30.6 ± 3.9	0.34	-0.43	0.40
AMAc (cm^2^)	34.4 (14.1-70.7)	42.9 (25.4-84.9)	<0.01^∗^	-8.55	38.1 ± 20.8	43.6 ± 17.5	0.02^∗^	-5.55	0.33
CP (cm)	35.5 ± 4.1	36.5 ± 2.8	0.13	-0.96	36.1 (29.8-42)	37.5 (30.3-59.8)	0.03^∗^	-1.45	0.79
BF (%)	39.7 ± 7.6	34.7 ± 6.7	<0.01^∗^	-5.01	37.6 ± 6.3	35.0 ± 5.7	<0.01^∗^	-2.62	0.02^a^
CRP (mg/mL)	1.6 (0.3-7.9)	1.7 (0.1-7.1)	0.75	-0.01	2.1 (0.2-8.0)	1.6 (0.3-6.1)	0.18	0.43	0.80
IL-8 (pg/mL)	256 (201.1-322.1)	251.2 (189.9-491.3)	0.78	4.80	264.8 (198.7-448.7)	278.4 (169.1-447.7)	0.49	-13.60	0.64
CCL-2 (pg/mL)	282.7 (74.5-950.7)	229.9 (27.9-551.5)	0.24	53	310.7 (54.3-896.3)	226 (59.0-744.1)	0.02^∗^	84.70	0.44
Leptin (pg/mL)	2783 ± 769.5	2415 ± 699.4	0.03^∗^	368	2714 ± 625.8	2393 ± 626.8	0.01^∗^	321	0.81

The categorization into groups was done according to the median of HEI-R (65.71). Δ: delta (initial-final). HEI-R: Revised Health Eating Index; BMI: body mass index; WC: waist circumference; HC: hip circumference; WHR: waist-to-hip ratio; AC: abdominal perimeter; AP: arm perimeter; AMAc: corrected arm muscle area; CP: calf perimeter; BF (%): percentage of body fat; CRP: C-reactive protein; IL-8: interleukin 8; CCL-2: monocyte chemotactic protein 1. ^∗^Difference between paired groups. ^a^Difference between unpaired groups. Data expressed as the mean ± standard deviation or median (minimum and maximum).

**Table 2:** Anthropometric and inflammatory variables, according to the dTAC and 19 wk intervention.

≤dTAC (*n* = 20)					>dTAC (*n* = 20)				*p* value
Variable	Baseline	Final	*p* value	Δ	Baseline	Final	*p* value	Δ	
Weight (kg)	75.2 ± 12.9	74.4 ± 12.4	0.24	0.87	68.8 ± 14.1	69.4 ± 13.8	0.44	-0.63	0.55
BMI (kg/m^2^)	28.8 ± 4.9	28.5 ± 4.6	0.24	0.35	26.4 ± 3.8	26.7 ± 3.6	0.45	-0.23	0.57
WC (cm)	91.6 ± 9.0	88.2 ± 9.4	0.01^∗^	3.36	87.2 ± 10.7	84.7 ± 10.5	<0.01^∗^	2.44	0.85
HC (cm)	101.9 ± 10.8	102.4 ± 9.4	0.80	-0.50	97.7 ± 8.3	98.3 ± 7.5	0.43	-0.59	0.43
WHR	0.90 ± 0.09	0.86 ± 0.08	0.05	0.04	0.89 ± 0.07	0.86 ± 0.06	<0.01^∗^	0.03	0.68
AC (cm)	95.3 ± 12.6	95.2 ± 11.5	0.94	0.09	91.3 ± 12.3	90.4 ± 13.4	0.57	0.87	0.82
AP (cm)	31.1 ± 3.5	32.1 ± 2.7	0.08	-0.94	29.2 ± 4.3	29.7 ± 4.0	0.28	-0.48	0.50
AMAc (cm^2^)	42.7 ± 17.0	49.9 ± 16.0	0.01^∗^	-7.19	31.8 (5.7-91.7)	32.7 (14.4-82.7)	<0.01^∗^	-0.86	0.98
CP (cm)	35.5 ± 4.0	36.6 ± 2.5	0.08	-1.17	35.9 (29.8-42.2)	37.5 (30.3-59.8)	0.05	-1.60	0.83
BF (%)	39.4 ± 8.1	34.5 ± 7.0	<0.01^∗^	4.86	37.9 ± 5.7	35.2 ± 5.3	<0.01^∗^	2.78	0.04^a^
CRP (mg/mL)	2.2 (0.2-8.0)	2.0 (0.1-7.1)	0.71	0.16	1.4 (0.2-8.0)	1.5 (0.3-6.1)	0.10	-0.05	0.06
IL-8 (pg/mL)	243.8 ± 34.2	263 ± 57.2	0.17	-19.20	280.4 (222.8-448.7)	275.2 (176.3-491.3)	0.54	5.20	0.20
CCL-2 (pg/mL)	282.6 ± 146	258.7 ± 137.4	0.60	23.90	338.7 (54.3-950.7)	244.7 (59.0-744.1)	<0.01^∗^	94	0.09
Leptin (pg/mL)	2593 ± 650.7	2303 ± 697.6	0.09	290	2904 ± 715.4	2508 ± 611.1	<0.01^∗^	396	0.60

The categorization into groups was done according to the median of dTAC (14.23). Δ: delta (initial-final). dTAC: total antioxidant dietary capacity; BMI: body mass index; WC: waist circumference; HC: hip circumference; WHR: waist-to-hip ratio; AC: abdominal perimeter; AP: arm perimeter; AMAc: corrected arm muscle area; CP: calf perimeter; BF (%): percentage of body fat; CRP: C-reactive protein; IL-8: interleukin 8; CCL-2: monocyte chemotactic protein 1. ^∗^Difference between paired groups. ^a^Difference between unpaired groups. Data expressed as the mean ± standard deviation or median (minimum and maximum).

## Data Availability

Data are available from https://www.repositorio.ufop.br/handle/123456789/12340.
